# Acute Colonic Pseudo-Obstruction with Feeding Intolerance in Critically Ill Patients: A Study according to Gut Wall Analysis

**DOI:** 10.1155/2017/9574592

**Published:** 2017-03-12

**Authors:** Chenyan Zhao, Tingbin Xie, Jun Li, Minhua Cheng, Jialiang Shi, Tao Gao, Fengchan Xi, Juanhong Shen, Chun Cao, Wenkui Yu

**Affiliations:** ^1^Research Institute of General Surgery, Jinling Hospital, No. 305 Zhongshan East Road, Nanjing 210002, China; ^2^Medical School, Nanjing University, No. 22 Hankou Road, Nanjing 210002, China; ^3^Southern Medical University, No. 1023 Shatai South Road, Guangzhou 510515, China; ^4^Jining No.1 People's Hospital, No. 99 Jianshe West Road, Jining 272000, China

## Abstract

*Objective*. To compare the differences between acute colonic pseudo-obstruction (ACPO) with and without acute gut wall thickening. *Methods*. ACPO patients with feeding tolerance were divided into ACPO with no obvious gut wall thickening (ACPO-NT) group and ACPO with obvious acute gut wall thickening (ACPO-T) group according to computed tomography and abdominal radiographs. Patients' condition, responses to supportive measures, pharmacologic therapy, endoscopic decompression, and surgeries and outcomes were compared. *Results*. Patients in ACPO-T group had a significantly higher APACHE II (11.82 versus 8.25, *p* = 0.008) and SOFA scores (6.47 versus 3.54, *p* < 0.001) and a significantly higher 28-day mortality (17.78% versus 4.16%, *p* = 0.032) and longer intensive care unit stage (4 versus 16 d, *p* < 0.001). Patients in ACPO-NT group were more likely to be responsive to supportive treatment (62.50% versus 24.44%, *p* < 0.001), neostigmine (77.78% versus 17.64%, *p* < 0.001), and colonoscopic decompression (75% versus 42.86%, *p* = 0.318) than those in ACPO-T group. Of the patients who underwent ileostomy, 81.25% gained benefits. *Conclusions*. ACPO patients with gut wall thickening are more severe and are less likely to be responsive to nonsurgical treatment. Ileostomy may be a good option for ACPO patients with gut wall thickening who are irresponsive to nonsurgical treatment.

## 1. Introduction

Feeding intolerance (FI) is a common and clinically important problem in critically ill patients. FI manifested as gastrointestinal symptoms such as abdominal distension, diarrhea, vomiting, and gastric retention, and inadequate enteral calorie intake is the biggest challenge in maintaining enteral nutrition (EN) in critically ill patients, which could cause or exacerbate malnutrition and has been associated with longer intensive care unit (ICU) stay and higher morbidity and mortality [1, 2]. Gastrointestinal dysfunctions are the major cause of FI. Most doctors and studies have focused on the upper gastrointestinal dysfunctions especially gastroparesis since they are more likely to present nausea, vomiting, and gastric retention; particularly, gastric residual volume (GRV) is the most frequently used parameter to monitor FI, whereas the lower gastrointestinal factors were often neglected [3, 4]. However, the stomach and small intestine may function properly, but FI still exists which could be the result of colonic dysfunction.

Acute colonic pseudo-obstruction (ACPO), also known as Ogilvie's syndrome, is characterized by acute dilation of the large bowel with obstructive symptoms in the absence of mechanical obstruction [5]. The remarkable changes in ACPO patients are the massive dilation and dysmotility of colon which could cause FI, and if left untreated, ACPO can lead to colonic necrosis and perforation [6]. In critical patients, we have observed an interesting phenomenon that patients who are feeding intolerant and have a great dilated large bowel are sometimes accompanied with a markedly thickening gut wall which could be detected by imaging test and those who are with acute gut wall thickening often have a poor outcome. In general, ACPO was mainly caused by an imbalance between parasympathetic and sympathetic innervation [7]. However, in critical patients, severe trauma, major surgery, sepsis, shock, or mesenteric vascular occlusion will cause great stress, intestinal ischemia/reperfusion, and acute inflammation in the gut wall, inducing acute edema in the colon which could be another reason for the dysfunction of the colon [8, 9].

Supportive treatment, cholinergic drugs, decompression, and surgery were usually sequentially used to treat patients with ACPO [5, 7]. However, to the best of our knowledge, there is no study distinguishing the two different ACPOs in terms of treatment. Studies have evaluated the responses of ACPO patients to these treatments, but got conflicting results. A retrospective study by Mehta et al. [10] enrolled 27 patients with ACPO who have received supportive treatment, and only eight (30%) of them achieved spontaneous resolution, which is contrary to that of Loftus et al. [11], who showed that a majority of patients with ACPO were responsive to supportive measures. Both of them have evaluated the predictive factors for response to neostigmine, finding that postoperative patients, females, or older-aged patients were more likely to be responder of neostigmine while the presence of electrolyte imbalance and antimotility medication use were the risk factors for poor response to neostigmine. However, none of them has taken the gut wall edema into consideration [10, 11]. Considering the different pathophysiologies and clinical courses of general ACPO and ACPO with acute gut wall thickening (edema), the recommended treatment protocol for ACPO may not be suitable for both of the two ACPOs.

Therefore, this study was designed to compare the outcomes of the two different ACPOs and their responses to different treatments, in order to alert doctors to distinguish the two ACPOs.

## 2. Methods

### 2.1. Patients and Design

This study was approved by the ethics committee of Jinling Hospital and the Medical School of Nanjing University, and the protocols were registered at ClinicalTrials.gov (NCT02939508). This is a prospective, single-center, observational study conducted at the 39-bed surgical intensive care unit (SICU) of the General Surgical Department of Jinling Hospital, affiliated to the Medical School of Nanjing University from July 1, 2014, to July 1, 2016. Patients in our ICU aged 18–75 years who had FI and diagnosed with ACPO were included. EN was started within 24–48 h after ICU admission if possible unless electively not fed by the attending doctors. FI was thought to be present if at least 50% of the calculated needs via enteral feeding could not be reached after 72 h EN attempt due to signs of nausea, vomiting, gastric retention (a single GRV > 250 ml), abdominal pain, abdominal distension, ileus or severe diarrhea, or no feeding because of any clinical conditions (active gastrointestinal hemorrhage, obstruction, intestinal necrosis, gastrointestinal fistula or perforation, and so forth). FI was not registered if the patient was electively not fed or if the enteral feeding was disrupted or withheld due to procedures [12, 13]. All patients with FI and fed via the jejunum pathway were screened, and those who were diagnosed with ACPO were included. ACPO was identified if acute dilation of the colon or cecum (colonic diameter > 6 cm or cecal diameter > 9 cm) was observed on computed tomography (CT) or abdominal radiography [5]. Patients with any of the following conditions were excluded: (1) mechanical gastrointestinal obstruction (including tumor and stercoral obstruction); (2) gastrointestinal hemorrhage within 72 hours before inclusion; (3) presence of intra-abdominal abscesses at inclusion; (4) presence of intestinal perforation, necrosis, or fistula at inclusion; (5) history of inflammatory bowel disease (ulcerative colitis or Crohn's disease) or radiation enteritis; (6) pregnancy; (7) contraindications of neostigmine administration; and (8) disconcerting with endoscopy or surgical treatment or treatment abandonment. The enrolled patients were divided into two groups according to the gut wall thickness (edema) on CT (ileocecus): ACPO with no obvious thickening (edema) of the colonic gut wall (ACPO-NT) group and ACPO with obvious acute thickening (edema) of the colonic gut wall (ACPO-T) group.

### 2.2. Treatment Protocol

**Figure 1:**
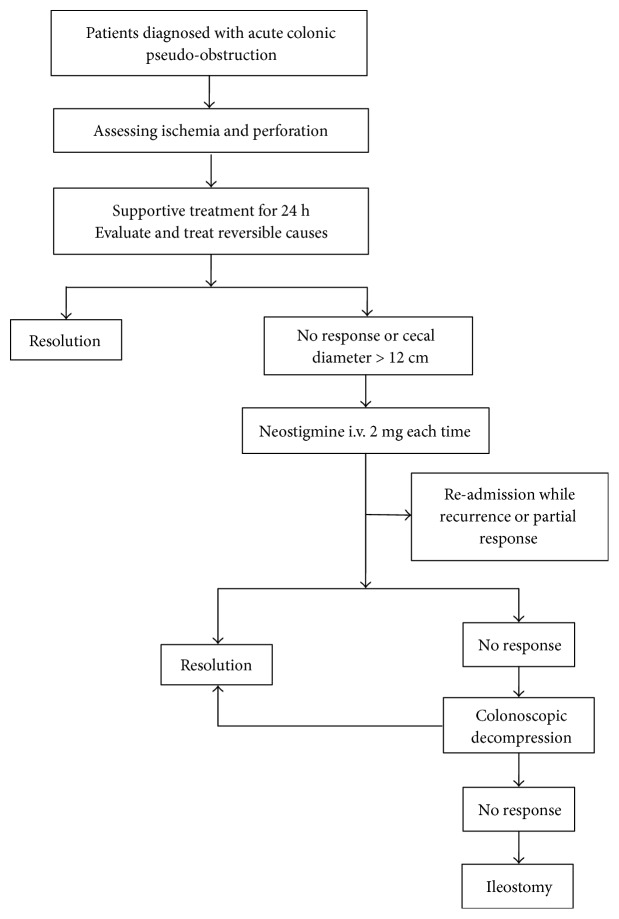
Treatment protocol.

Once the patients had confirmed diagnosis of ACPO, they started the treatment according to our protocol depicted in Figure 1. Specific processes were as follows:

Supportive measures: Firstly, hydro-electrolyte and thyroid function were assessed and corrected if abnormal. Blood glucose was maintained within normal levels. Patients prone to sepsis were administered antibiotics empirically and adjusted to more targeted antibiotics according to the blood culture and drug sensitivity test results. Patients were subject to fasting and gastrointestinal decompression via stomach and anal tubes. GRV was assessed four times a day. Pharmaceuticals affecting bowel movement, such as opiates, anticholinergic drugs, and calcium channel blockers, were suspended as quickly as possible.

Pharmacologic therapy with cholinergic drugs: Intravenous administration of neostigmine was considered if the cecal diameter was >10 cm after applying the treatment described above without signs of amelioration within 24 hours or cecal diameter of >12 cm [14, 15].

Colonoscopic decompression: This procedure was applied when obvious cecum distension was present (diameter > 10 cm) for more than 3 days and when there were no signs of improvement after 24–48 hours of supportive or neostigmine treatment or if neostigmine was contraindicated [16]. Colonoscopic decompression was performed by 2 experienced endoscopy physicians at the bedside or at an endoscopy room.

Surgical intervention: Surgery was indicated when colonic distension lasted more than 6 days or obvious cecal distention (diameter > 10 cm) continued after 48–72 hours of supportive or pharmacologic management and colonoscopic decompression [17]. Ileostomy was performed under epidural or local anesthesia by a team of experienced surgeons. If the patients had colonic necrosis or perforation, ileostomy was superseded by colectomy under general anesthesia [18].

### 2.3. Date Collection

The following data were recorded: (1) demographic data including age, sex, and body mass index (BMI) at grouping; (2) primary diagnosis, reasons for intensive care, and interventions before or at grouping; and (3) severity of illness, as assessed by the Acute Physiology and Chronic Health Evaluation II (APACHE II) and Sequential Organ Failure Assessment (SOFA) scores within 24 hours from the time of grouping.

### 2.4. Follow-Up

After ICU admission, gastrointestinal dysfunction symptoms such as FI, vomiting, abdominal distension, and defecation were recoded every day. GRV and intra-abdominal pressure (IAP) which was reflected by bladder pressure were measured four times a day after ICU admission. Abdominal CTs were performed on days 1, 3, 5, and 7 and every week after inclusion, unless required more frequently because of an additional illness. Colonic and cecal diameters were evaluated independently by two trained fellows who were blinded to this study. Responses to treatment (supportive treatment, neostigmine, colonoscopy, and surgery) and colonic recovery time were also assessed and recorded through vital signs, laboratory tests, and CT examination.

Colonic recovery was identified when meeting the following criteria: (1) EN reached or surpassed 50% of the targeted needs during 3 days or longer and (2) evident improvement of colonic distension (colonic diameter < 6 cm or cecum diameter < 9 cm) or edema of the colonic wall shown on the abdominal CT [16]. Recurrence was defined as patients diagnosed with recurrent ACPO within 72 hours of recovery after a certain treatment.

### 2.5. End Points

The primary outcome was the 28-day mortality of patients in the two groups. Secondary outcomes included ICU mortality, hospitalization mortality, and the duration of the stay in the ICU and in the hospital of the two groups. Complications of the two groups such as intra-abdominal hypertension (IAH; IAP is found to be 12 mmHg or higher, confirmed by at least two measurements); abdominal compartment syndrome (ACS; IAP above 20 mmHg with new onset organ failure confirmed by minimally two standardized measurements); sepsis; gastrointestinal hemorrhage; colonic perforation; colonic necrosis; and new organ dysfunction were also recorded.

### 2.6. Statistical Analysis

Statistical analysis was performed with SPSS 20.0 (SPSS, Inc., an IBM Company, Chicago, IL). Categorical variables were compared using the *χ*^2^ test or Fisher's exact test. The parametric tests will be applied when normality (and homogeneity of variance) assumptions are satisfied; otherwise, the equivalent nonparametric test will be used. Parametric tests were conducted using *t*-tests, and nonparametric tests were conducted using the Mann-Whitney *U* test. Multivariate analysis by binary logistic regression was done for the risk of 28-day mortality and failure of nonsurgical treatment. A 2-tailed *p* < 0.05 was considered as statistically significant.

## 3. Results

### 3.1. General Information

**Figure 2:**
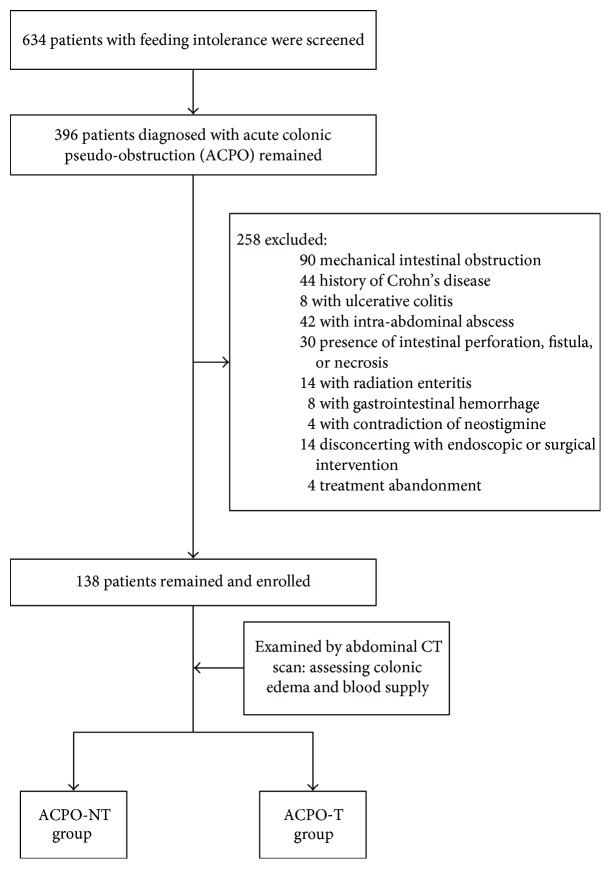
The screening and grouping protocol.

From July 1, 2014, to July 1, 2016, 634 patients were enrolled for FI via the jejunum pathway. Of these, 396 patients remained due to the diagnosis of ACPO. Of the remaining 396 subjects, 258 patients were excluded: 90 with mechanical intestinal obstruction; 44 with history of Crohn's disease; 8 with ulcerative colitis; 42 with intra-abdominal abscess; 30 with intestinal perforation, fistula, or necrosis at inclusion; 14 with radiation enteritis; 8 with gastrointestinal hemorrhage within 72 h; 4 with contradiction of neostigmine; 14 disconcerting with endoscopic or surgical intervention (patients or their families); and 4 treatment abandonment. Thus, 138 patients (32 men and 106 women) were studied. The subjects were classified into two groups, the ACPO-NT group (*n* = 48) and ACPO-T group (*n* = 90), according to colon conditions. The process of patient screening and grouping is presented in Figure 2.

### 3.2. Comparison of the General Data between the ACPO-NT Group and the ACPO-T Group

There were no differences in sex ratio (*p* = 0.224) and BMI (*p* = 0.536) between the two groups. The mean age was significantly lower in the ACPO-NT group (*p* = 0.007). However, APACHE II (*p* = 0.008) and SOFA scores (*p* < 0.001), mechanical ventilation (*p* < 0.001), sepsis incidence (*p* = 0.004), and vasoactive drug usage (*p* = 0.004) were significantly higher in the ACPO-T group. Details are listed in Table 1.

### 3.3. Comparison of the Gastrointestinal Dysfunction Parameters at the Time of Grouping between the ACPO-NT Group and the ACPO-T Group

Although the occurrence of vomiting (*p* = 0.356), abdominal distension (*p* = 0.192), IAP (*p* = 0.671), and days without defecation (*p* = 0.607) showed no statistical difference between the groups, the incidence of gastric retention (*p* = 0.002) and GRV (*p* = 0.002) were significantly higher in the ACPO-T group during the 28-day period. The colonic diameters (*p* = 0.110) and cecal diameters (*p* = 0.853) were not statistically different at grouping between the groups as Table 2 shows.

### 3.4. Comparison of the Treatment Responses

The overall efficacy of the nonsurgical treatment in the ACPO-NT group reached 97.91% versus 64.4% in the ACPO-T group (*p* < 0.01). Patients in the ACPO-NT group had significantly higher efficacy in conservative treatment (62.50% versus 24.44%, *p* < 0.001). Neostigmine efficacy was only 17.64% in the ACPO-T group, which was significantly lower than that in the ACPO-NT group (77.78%, *p* < 0.001). Colonoscopic decompression had better results in the ACPO-NT group than in the ACPO-T group, though with no significant difference (75% versus 42.86%, *p* = 0.318). One patient in the ACPO-NT group irresponsive to nonsurgical treatment underwent ileostomy and eventually recovery, while 32 in ACPO-T group irresponsive to nonsurgical treatment underwent ileostomy, colectomy, or colostomy, which were also effective in 26 subjects as Table 3 shows.

### 3.5. Comparison of Outcomes between the Two Groups

Within 28 days, 16 (17.78%) patients died in the ACPO-T group and 2 (4.17%) deaths occurred in the ACPO-NT group (*p* = 0.032). During the ICU period, 18 patients died (20.00%) in the ACPO-T group and 1 (2.08%) death occurred in the ACPO-NT group, with a significant difference (*p* = 0.003). During hospitalization, 20 (22.22%) patients died in the ACPO-T group versus 3 (6.25%) in the ACPO-NT group (*p* = 0.017). Median ICU stay was significantly shorter in the ACPO-NT group than in the ACPO-T group (4 versus 16 d, *p* < 0.001). Similarly, the ACPO-NT group has significantly less median hospitalization days (15 versus 36 d, *p* < 0.001) (Table 4).

The incidence of IAH (*p* = 0.008) and ACS (*p* < 0.001) was significantly higher in the ACPO-T group than in the ACPO-NT group. During treatment process, subjects diagnosed with sepsis were 58 (64.44%) in the ACPO-T group and 12 (25.00%) in the ACPO-NT group (*p* < 0.001). Two (4.17%) patients suffered gastrointestinal hemorrhage in the ACPO-NT group versus 8 (8.89%) in the ACPO-T group (*p* = 0.494). No colonic perforation or necrosis occurred in the ACPO-NT group while 6 (6.67%) patients in the ACPO-T group had colonic perforation and 4 (4.44%) had colonic necrosis, though showing no statistical difference (*p* = 0.092 and *p* = 0.298, resp.). The ACPO-T group had a significantly higher rate of new organ dysfunction than the ACPO-NT group (*p* < 0.001) (Table 4).

**Figure 3:**
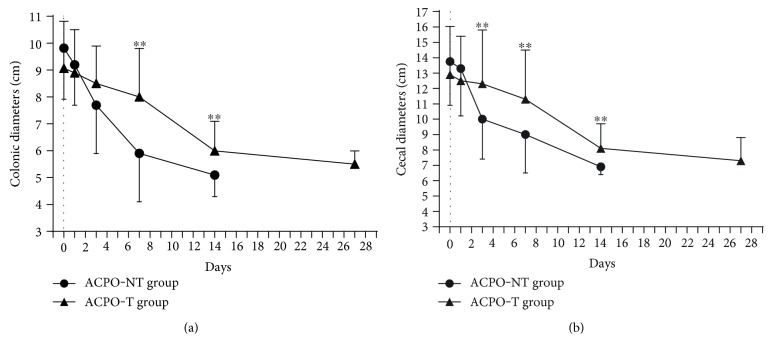
Colonic and cecal diameters in the CT scan at grouping and after 1, 3, 7, 14, and 28 days of treatment. (a) Changes of colonic diameter. On days 7 and 14, the colonic diameter of the ACPO-NT group was significantly less than that of the ACPO-T group. (b) Changes of cecal diameter. On days 3, 7, and 14, the cecal diameter of the ACPO-NT group showed a significant reducing rate, ^∗∗^*p* < 0.01.

At the time of grouping, there was no significant difference in colonic or cecal diameter between the two groups (*p* = 0.110 and *p* = 0.853, resp.). After treatment, colonic and cecal diameters of both groups showed a decreasing trend. On days 7 and 14, the colonic diameter of the ACPO-NT group was significantly less than that of the ACPO-T group. Similarly, on days 3, 7, and 14, the cecal diameter of the ACPO-NT group showed a significant reduction rate (Figure 3).

### 3.6. Subgroup Analysis of Ileostomy

**Figure 4:**
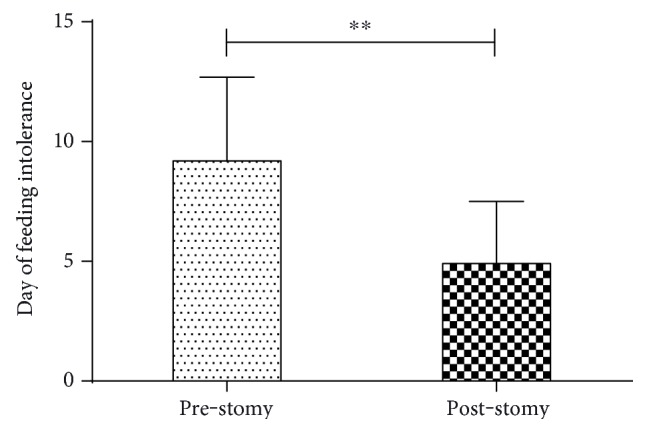
Comparison of the preoperative and postoperative days of feeding intolerance in patients who received ileostomy, colectomy, or colostomy. After surgery, patients had significantly less days of feeding intolerance, ^∗∗^*p* < 0.01.

In the ACPO-NT group, only one patient underwent ileostomy because of irresponsiveness to nonsurgical treatment, whereas 32 patients in the ACPO-T group underwent ileostomy, colectomy, or colostomy. Of those, 6 patients showed colonic redilation (18.75%), which was significantly lower than that (62.5%) observed preoperatively (*p* = 0.013). Among the 32 surgery-treated patients in the ACPO-T group, 10 underwent colectomy or colostomy because of colonic ischemia (*n* = 4) or perforation (*n* = 6); 22 subjects received ileostomy because of the ineffectiveness of conservative and colonoscopic treatments or because of recurrence; and 2 subjects underwent ileostomy due to colonic dilation after colectomy or colostomy. The postoperative feeding intolerance time was significantly shorter than the preoperative time (*p* < 0.01), as shown in Figure 4. The postoperative mortality (4 after colectomy, 2 after colostomy; 18.75%) showed no significant difference compared with the total mortality (22.2%) in the ACPO-T group (*p* = 0.212).

### 3.7. Multivariate Analysis for the Risk of 28-Day Mortality and Failure of Nonsurgical Treatment

The results of multivariate analysis are shown in Table 5. Age (OR 1.04, *p* = 0.031), APACHE II (OR 1.03, *p* = 0.006), SOFA (OR 1.06, *p* < 0.001), mechanically ventilated (OR 1.53, *p* = 0.026), receiving vasoactive drugs (OR 2.14, *p* = 0.018), and the presence of acute gut wall thickening (edema) (OR 1.14, *p* = 0.047) were independent risk factors for the 28-day mortality, while for the failure of nonsurgical treatment, APACHE II (OR 1.05, *p* = 0.029), SOFA (OR 1.08, *p* < 0.001), and the presence of acute gut wall thickening (edema) (OR 1.51, *p* < 0.001) were detected to be the independent risk factors.

## 4. Discussion

Our results displayed that ACPO patients with acute gut wall thickening (edema) were more severe than those without gut wall thickening (edema), with higher ICU scores, longer FI days, more frequent shock state, more frequent perforation or necrosis, more frequent presence of ACS and Multiple Organ Dysfunction Syndrome (MODS), and finally, longer ICU stay and higher mortality rates. Regarding the treatment responses, nonsurgical treatments (supportive treatment, neostigmine administration, or endoscopic decompression) were more likely to be effective for ACPO patients without gut wall edema, while ileostomy could be an effective surgical treatment for ACPO patients with gut wall thickening. Multivariate analysis also revealed that acute thickening of the colonic wall is a risk factor for the 28-day mortality and failure of nonsurgical treatment in ACPO patients.

Up until now, the incidence of ACPO remains unclear. Vanek and Al-Salti reviewed 400 cases and found that most studied ACPO patients have underwent retroperitoneal or spine surgery or had cerebral, spine, or pelvic trauma, and other conditions like imbalance of electrolyte and infection could also cause ACPO [19]. General ACPO is considered to be the result of colonic autonomic dysregulation with decreased activity of parasympathetic and increased activity of sympathetic stimulation. The neurotransmitters which can stimulate colonic movement mainly include acetylcholine, neurokinin A, and substance P, while the inhibitory nerves express vasoactive intestinal polypeptide and nitric oxide [6]. Thus, administration of cholinergic drugs such as neostigmine which acts as a reversible acetylcholinesterase inhibitor can achieve good results. Those patients usually have good general condition, having transient ischemia and hypoxemia or mild systemic and local inflammation. However, in our practice, sepsis, shock, or intestinal ischemia/reperfusion induced acute inflammation and edema in the gut wall will also cause gastrointestinal dysfunction and colonic dilation. This could be the other type of ACPO; here, we call it “critical illness-associated ACPO (CIACPO).” In the present study, we divided our patients into the ACPO-NT and ACPO-T groups to represent the conventional ACPO and critical illness-associated ACPO. We found that critical illness-associated ACPO patients were more likely to suffer shock, ACS, and MODS. The explanations must be related to its pathophysiology.

The gastrointestinal tract comprises a series of important and complex functions, including digestion, absorption, endocrine, and mechanical and immune barriers [20, 21]. As the biggest immune organ of the body, and the biggest pool of bacteria, acute gastrointestinal dysfunction has been considered to be the motor of MODS in critical patients [22, 23]. CIACPO is one of the acute gastrointestinal dysfunctions (AGID) mainly induced by sepsis or ischemia/reperfusion. Contrary to general ACPO in which the main impairment of the gut is motility caused by dysregulation of the colonic nervous system [7], multiple barrier dysfunctions are happening in AGID [24]. Animal studies have shown that, in sepsis or intestinal ischemia/reperfusion models, hyperpermeability of the intestinal mucosal barrier and vascular as well as microcirculation dysfunction could cause a massive leakage of intravascular fluid and albumin into the interstitial space causing edema of the gut wall or into the enteric lumen causing diarrhea or abdominal distension, which could be one of the reasons for more frequent ACS or shock [9, 25, 26]. ACS and shock could then greatly impact the perfusion of organs, thereby leading to MODS [27]. On the other hand, the change of intraluminal environment and dysmotility will cause intestinal flora disturbance and toxin release [24]. With the hyperpermeability of the intestinal mucosal barrier and vascular endothelium, the disturbed bacteria and their toxin will easily translocate into the circulation, aggravating Systemic Inflammatory Response Syndrome (SIRS) and sepsis and inducing septic shock and MODS.

The remission rates of nonsurgical treatments including supportive treatment, neostigmine administration, and endoscopic decompression have been reported with a wide range of 70–90%, 60–100%, and 60–90%, respectively [5]. Elsner et al. showed that the recovery rate of colonic pseudo-obstruction from nonsurgical treatment was 70–85%, and the rest required surgical intervention although at a different percentage [28]. In our study, 47 out of 48 (97.91%) patients in the ACPO-NT group recovered from sequential nonsurgical treatments, while in the ACPO-T group, the nonsurgical recovery rate was only 64.4%. The efficiency of nonsurgical intervention in ACPO-T was much lower than that in ACPO-NT (17.64% versus 62.5% and 42.86% versus 75% for neostigmine and colonoscopy, resp.). We thought that the wide range of efficiency of nonsurgical treatments could be attributed to the indiscrimination of the two different ACPOs. As for patients with acute colonic edema, colonic dysfunction was caused by inflammation and colonic edema instead of autonomic regulation dysfunction; actually, those patients easily progressed to ACS [29] as shown in our results, which could be the reason why they were less responsive to neostigmine than in the ACPO-NT group. Meanwhile, endoscopic decompression could aspirate gas and fluid in the colon, but the inflammation and edema would not subside in the short term, thus delaying the recovery. Like gastroparesis, the gastrointestinal decompression could relieve the symptom, but the recovery still required time.

At present, surgery is the last resort for ACPO when nonsurgical intervention is irresponsive or colonic ischemia and perforation occurs [17]. Measures such as colectomy, colostomies, and cecostomy are commonly applied [30]. A study by Vanek and Al-Salti [19] which showed that among the 129 ACPO patients who have received an ostomy, successful decompression for tube cecostomy, cecostomy, and ileostomy or colostomy was achieved in 100%, 95%, and 73%, respectively. Though cecostomy seemed to be more likely to achieve better outcomes than ileostomy or colostomy for conventional ACPO patients, we chose to apply ileostomy in our study if the nonsurgical treatment failed to take CIACPO into consideration. Interestingly, ileostomy mitigated the postoperative FI (62.5% preoperatively versus 23.08% postoperatively, *p* = 0.013) and reduced the dilation of the large bowel and recurrence of nonmechanical obstruction significantly.

Other research has indicated that terminal ileum could be the main place where bacterial translocation occurs, due to ileocecal reflux under intestinal ischemia/reperfusion conditions [31, 32]. Therefore, ileostomy may prevent bacterial translocation besides having the merits of minimal trauma. On the other hand, since nutrition was mainly absorbed in the small bowels [33], ileostomy could make the large bowels rest and EN could be avoided in the large bowel, thus avoiding the occurrence of FI caused by colonic dysfunction which may also be secondary to colostomy or colectomy; thus, not surprisingly, ileostomy could avoid the occurrence of FI afterwards. For these reasons, patients in this study all underwent ileostomy when surgical interventions were needed, and colectomy was only applied in cases of colonic necrosis and perforation.

There are still some limitations in our study. Firstly, abdominal CT was an important measurement for colon lesion in our study. Although abdominal CT had a distinct advantage over other examinations to assess colonic edema and dilation, there were no unified criteria or scale system to evaluate intestinal lesions. Misjudgment was possible, even though two experienced radiologists viewed the CT independently in our study. Because our SICU focuses on gastrointestinal surgery, our data and management experience of AGID may not fit the critical patients in other ICUs completely. Secondly, the sample size is relatively small especially for the ACPO-NT group; thus, the significance between the groups may not be detected in some aspects. Future investigations based on a large survey sample are needed. Thirdly, we only used ileostomy as the last resort for treatment. Other surgical methods should also be applied to make a comparison with each other. Thirdly, in the process of screening patients, we also found that some patients with colonic gut wall edema but without colonic dilation already had colonic dysfunction and FI. Thus, it is probable to speculate that ACPO with gut wall edema is proceeded by or is a variant of it. As a matter of fact, critical illness-related ileus was introduced by van der Spoel et al. in 2001 [34], though they have not included patients with colonic dilation or mentioned the gut wall edema. In that case, comparison between colonic edema with dilation and colonic edema without dilation may be necessary, too, in future.

In conclusion, acute gut wall thickening (edema) is a risk factor for failure of nonsurgical treatment in ACPO patients and is associated with worse outcome than ACPO patients without acute gut wall thickening (edema). For ACPO patients with acute gut wall thickening (edema), ileostomy could be a good surgical method to relieve the symptoms and to enhance the recovery of ACPO. This study is the first to discriminate the two different ACPOs, to discuss the different pathophysiologies, and to provide evidence for treatment.

## Figures and Tables

**Table 1:** Characteristics of the study population.

Characteristics	ACPO-NT (*N* = 48)	ACPO-T (*N* = 90)	*p*
Age (year)^∗^	48.38 ± 14.47	57.16 ± 11.46	0.007
Sex, male, *n* (%)^†^	14 (29.2)	18 (20.0)	0.224
BMI^∗^	23.73 ± 2.32	23.33 ± 2.65	0.536
APACHE II^∗^	8.25 ± 5.04	11.82 ± 5.22	0.008
SOFA^∗^	3.54 ± 3.04	6.47 ± 3.04	<0.001
Primary diagnosis, *n* (%)^†^			
Abdominal/multitrauma	22 (45.83)	18 (20.00)	
Vascular surgery	10 (20.83)	4 (4.44)	
Pancreatic surgery	4 (8.33)	6 (6.67)	
Gastrointestinal surgery	2 (4.17)	12 (13.33)	
SAP	0 (0.00)	14 (15.56)	
Biliary surgery	0 (0.00)	8 (8.89)	
Peritonitis	0 (0.00)	8 (8.89)	
Hepatic surgery	0 (0.00)	6 (6.67)	
Others	10 (20.83)	4 (4.44)	
Reason for intensive care, *n* (%)^†^			
IAH/ACS	18 (37.50)	24 (26.67)	
Sepsis	10 (20.83)	20 (22.22)	
Gastrointestinal hemorrhage	4 (8.33)	8 (8.89)	
Intra-abdominal hemorrhage	4 (8.33)	6 (6.67)	
Acute renal failure	2 (4.17)	12 (13.33)	
Others	8 (16.67)	2 (2.22)	
Mechanically ventilated, *n* (%)^†^	10 (20.83)	56 (62.22)	<0.001
Sepsis, *n* (%)^†^	6 (12.50)	32 (35.56)	0.004
Receiving vasoactive drugs, *n* (%)^†^	6 (12.50)	32 (35.56)	0.004
Receiving IV opioid, *n* (%)^†^	18 (37.50)	46 (51.11)	0.127
Receiving pharmacologic paralysis, *n* (%)^†^	4 (8.33)	12 (13.33)	0.578

ACPO-NT: acute colonic pseudo-obstruction without obvious thickening of the colonic gut wall; ACPO-T: acute colonic pseudo-obstruction with obvious acute thickening of the colonic gut wall; BMI: body mass index; APACHE II: Acute Physiology and Chronic Health Evaluation II; SOFA: Sequential Organ Failure Assessment; SAP: severe acute pancreatitis; IAH/ACS: intra-abdominal hypertension/abdominal compartment syndrome.

^∗^Values are expressed as mean ± SD; ^†^values are expressed as *n* (%).

**Table 2:** Comparison of the gastrointestinal dysfunction-related parameters at the time of grouping between the groups.

Characteristics	ACPO-NT (*N* = 48)	ACPO-T (*N* = 90)	*p*
Gastric retention, *n* (%)^‡^	6 (12.50)	34 (37.78)	0.002
GRV (ml)^†^	133 (53–285)	288 (76–507)	0.002
IAP (mmHg)^∗^	9.38 ± 6.84	10.01 ± 5.31	0.671
Colonic diameter (cm)^∗^	9.82 ± 1.9	9.07 ± 1.75	0.110
Cecal diameter^∗^	13.75 ± 2.85	12.9 ± 3.14	0.853
Vomiting, *n* (%)^‡^	8 (16.67)	10 (11.11)	0.356
Abdominal distention, *n* (%)^‡^	20 (41.67)	48 (53.33)	0.192
Without defecation for 3 days, *n* (%)^‡^	38 (79.17)	46 (51.11)	0.001
Time span to last defecation (day)^∗^	4.04 ± 2.18	4.31 ± 2.00	0.607

ACPO-NT: acute colonic pseudo-obstruction without obvious thickening of the colonic gut wall; ACPO-T: acute colonic pseudo-obstruction with obvious acute thickening of the colonic gut wall; GRV: gastric residual volume; IAP: intra-abdominal pressure.

^∗^Values are expressed as mean ± SD; ^†^values are expressed as median (range); ^‡^values are expressed as *n* (%).

**Table 3:** Comparison of the efficacy of nonsurgical treatment and ileostomy between the groups.

Characteristics	All	ACPO-NT	ACPO-T	*p*
Nonsurgical treatment	105/138 (73.91)	47/48 (97.91)	58/90 (64.4)	<0.001
Conservative treatment	52/138 (37.68)	30/48 (62.50)	22/90 (24.44)	<0.001
Neostigmine	26/86 (30.23)	14/18 (77.78)	12/68 (17.64)	<0.001
Colonoscopic decompression	27/59 (45.00)	3/4 (75.00)	24/56 (42.86)	0.318
Surgery	27/33 (81.81)	1/1 (100)	26/32 (81.25)	0.212

ACPO-NT: acute colonic pseudo-obstruction without obvious thickening of the colonic gut wall; ACPO-T: acute colonic pseudo-obstruction with obvious acute thickening of the colonic gut wall.

All values are expressed as effective/total (efficacy %).

**Table 4:** Comparison of outcomes.

	ACPO-NT (*N* = 48)	ACPO-T (*N* = 90)	*p*
ICU mortality, *n* (%)^∗^	1 (2.08)	18 (20.00)	0.003
Hospitalization mortality, *n* (%)^∗^	3 (6.25)	20 (22.22)	0.017
28-day mortality, *n* (%)^∗^	2 (4.16)	16 (17.78)	0.032
ICU stage (day)^†^	4 (3, 7)	16 (11, 25)	<0.001
Hospitalization (day)^†^	15.00 (14, 20)	36.00 (23, 46)	<0.001
Complications			
IAH, *n* (%)^∗^	22 (45.83)	62 (68.89)	0.008
ACS, *n* (%)^∗^	4 (8.33)	34 (37.78)	<0.001
Sepsis, *n* (%)^∗^	12 (25.00)	58 (64.44)	<0.001
Gastrointestinal hemorrhage, *n* (%)^∗^	2 (4.17)	8 (8.89)	0.494
Colonic perforation, *n* (%)^∗^	0 (0.0)	6 (6.67)	0.092
Colonic necrosis, *n* (%)^∗^	0 (0.0)	4 (4.44)	0.298
New organ dysfunction, *n* (%)^∗^	4 (8.33)	36 (40.00)	<0.001

ACPO-NT: acute colonic pseudo-obstruction without obvious thickening of the colonic gut wall; ACPO-T: acute colonic pseudo-obstruction with obvious acute thickening of the colonic gut wall; ICU: intensive care unit; IAH: intra-abdominal hypertension; ACS: abdominal compartment syndrome.

^∗^Values are expressed as *n* (%); ^†^values are expressed as median (interquartile range).

All values were calculated from the time of grouping.

**Table 5:** Multivariate analyses of the possible risk factors for the 28-day mortality and failure of nonsurgical treatment.

	28-day mortality			Failure of nonsurgical treatment		
	OR	95% CI	*p* value	OR	95% CI	*p* value
Age (per year)	1.04	1.02–1.13	0.031	0.93	0.65–1.56	0.095
Sex (male)	1.26	0.79–1.54	0.298	1.02	0.84–1.23	0.302
BMI (kg/m^2^)						
18.5–25	1 (reference)			1 (reference)		
<18.5	1.16	0.82–1.91	0.247	1.08	0.73–1.48	0.495
>25	0.87	0.53–1.03	0.064	0.96	0.57–1.28	0.251
APACHE II (per point)	1.04	1.01–1.11	0.006	1.05	1.01–1.23	0.029
SOFA (per point)	1.06	1.02–1.17	<0.001	1.08	1.03–1.14	<0.001
Mechanically ventilated	1.53	1.24–2.06	0.026	1.25	0.93–1.92	0.060
Receiving vasoactive drugs	2.14	1.55–2.76	0.018	1.09	0.89–1.58	0.081
Receiving IV opioid	0.96	0.46–1.25	0.213	0.83	0.33–3.48	0.607
Acute gut wall thickening (edema)	1.14	1.03–1.29	0.047	1.51	1.27–1.69	<0.001
Receiving pharmacologic paralysis	1.02	0.83–2.31	0.365	0.99	0.72–2.27	0.212

OR: odds ratio; CI: confidence interval; BMI: body mass index; APACHE II: Acute Physiology and Chronic Health Evaluation II; SOFA: Sequential Organ Failure Assessment.
